# Reflective versus predictive value of urinary podocin, nephrin, and their ratio in diabetic kidney disease: a 12-month retrospective cohort study

**DOI:** 10.3389/fneph.2025.1681679

**Published:** 2026-01-21

**Authors:** Pringgodigdo Nugroho, Jeremia Siregar, Tri Juli Edi Tarigan, Kuntjoro Harimurti, Aida Lydia, Evy Yunihastuti, Pradana Soewondo, Hamzah Shatri

**Affiliations:** 1 Division of Nephrology and Hypertension, Department of Internal Medicine, Dr. Cipto Mangunkusumo National General Hospital, Faculty of Medicine Universitas Indonesia, Jakarta, Indonesia; 2 Department of Internal Medicine, Faculty of Medicine, Universitas Pelita Harapan, Tangerang, Indonesia; 3 Division of Endocrinology, Metabolism, and Diabetes, Department of Internal Medicine, Dr. Cipto Mangunkusumo National General Hospital, Faculty of Medicine Universitas Indonesia, Jakarta, Indonesia; 4 Division of Geriatrics, Department of Internal Medicine, Dr. Cipto Mangunkusumo National General Hospital, Faculty of Medicine Universitas Indonesia, Jakarta, Indonesia; 5 Division of Allergy and Clinical Immunology, Department of Internal Medicine, Dr. Cipto Mangunkusumo National General Hospital, Faculty of Medicine Universitas Indonesia, Jakarta, Indonesia; 6 Division of Psychosomatic and Palliative Medicine, Department of Internal Medicine, Dr. Cipto Mangunkusumo National General Hospital, Faculty of Medicine Universitas Indonesia, Jakarta, Indonesia

**Keywords:** albuminuria, biomarkers, diabetic kidney disease, eGFR, nephrin, podocin, podocin-nephrin ratio

## Abstract

**Introduction:**

Podocyte injury plays a central role in the development of diabetic kidney disease (DKD). Urinary podocin, nephrin, and the podocin–nephrin ratio (PNR) have been proposed as early indicators of glomerular injury, but their prognostic value remains uncertain. This study aimed to evaluate whether baseline urinary podocyte biomarkers reflect current disease severity and predict DKD progression.

**Methods:**

We conducted a retrospective cohort study involving 119 adults with type 2 diabetes and DKD. Baseline urinary podocin, nephrin, and PNR were measured using ELISA. Kidney outcomes were assessed over 12 months. DKD progression was defined as ≥5 mL/min/1.73 m² decline in estimated glomerular filtration rate (eGFR) and/or ≥30% increase in urine albumin-creatinine ratio (uACR). Follow-up uACR data were available for 52 participants. ROC analyses evaluated predictive performance.

**Results:**

At baseline, median eGFR was 68.1 mL/min/1.73 m² and median uACR was 112 mg/g. Over 12 months, eGFR declined significantly, while uACR showed high variability without consistent change. Among participants with complete outcome data, 19 (36.5%) experienced eGFR decline and 17 (32.7%) showed uACR progression. Baseline podocin, nephrin, and PNR were numerically higher in progressors but showed no significant group differences (all p > 0.3). Predictive performance was poor: AUCs for eGFR decline were 0.504 (podocin), 0.512 (nephrin), and 0.523 (PNR). For albuminuria progression, AUCs were 0.563, 0.524, and 0.544, respectively. Subgroup analyses similarly showed no significant predictive value.

**Discussion:**

These results suggest that single baseline measurements of podocin, nephrin, or PNR may have limited short-term prognostic value in DKD. However, the presence of these markers, even in patients with only moderate disease, supports their role as early indicators of podocyte stress.

**Conclusion:**

While urinary podocyte-associated proteins reflect early glomerular injury, their utility as stand-alone prognostic biomarkers over a one-year period may be limited. Larger longitudinal studies assessing biomarker trajectories and integrating additional molecular markers are warranted.

## 1 Introduction

Diabetic kidney disease (DKD) is a leading cause of end-stage kidney failure globally, resulting from chronic microvascular damage due to diabetes. It is characterized by progressive albuminuria and a declining glomerular filtration rate (GFR) (1). Early identification of individuals at risk of DKD progression is essential to enable timely intervention and delay irreversible nephron loss. While microalbuminuria remains the conventional early marker of DKD, it has recognized limitations. Albuminuria is not specific to DKD—it can occur in other conditions such as hypertension or obesity—and its course is not invariably progressive; some patients may revert to normoalbuminuria with therapy (2, 3). Thus, more specific and sensitive biomarkers are needed to both reflect early glomerular injury and predict future deterioration in renal function.

Emerging evidence highlights the role of podocyte injury as an early and central event in DKD pathogenesis. Podocytes are terminally differentiated cells crucial for maintaining the glomerular filtration barrier. Podocyte injury represents an early pathological event in DKD, characterized by cytoskeletal disruption, foot process effacement, and eventual detachment of podocytes from the glomerular basement membrane. It involves diverse pathways, including hyperglycemia, leading to oxidative stress, mitochondrial dysfunction, inflammation, and altered signaling. Injury or detachment of these cells contributes directly to proteinuria and glomerulosclerosis (4, 5).

Two podocyte-specific slit diaphragm proteins—nephrin and podocin—have garnered attention as potential noninvasive urinary biomarkers of podocytopathy. Nephrin is essential for maintaining selective glomerular permeability, and its urinary excretion (nephrinuria) has been observed in diabetic patients even before the onset of albuminuria (6). Studies have shown that nephrinuria correlates positively with albuminuria and negatively with GFR, indicating its potential to reflect early glomerular damage and predict DKD progression (3, 4, 7).

Podocin, a key structural protein localized at the podocyte foot processes, becomes detectable in the urine following such injury (8, 9). Urinary podocin levels have been found to correlate with reduced estimated GFR (eGFR) and with adverse metabolic parameters, including elevated HbA1c, blood pressure, and triglycerides (10–12). These findings support its potential as a marker of glomerular injury and DKD severity. Recent studies by Nugroho et al. further support the role of podocyte-associated biomarkers in DKD, demonstrating significant correlations between urinary nephrin and markers of endothelial dysfunction and disease activity, suggesting that nephrin may reflect earlier podocyte stress than podocin in DKD (13, 14).

Beyond individual markers, composite indices such as the urinary podocin-to-nephrin ratio (PNR) have been proposed to provide additional insight into the nature and stage of podocyte injury (15). Elevated PNR has been associated with active glomerular damage and worse renal outcomes in several glomerular diseases (16, 17). In DKD specifically, higher urinary PNR has been linked to increased tubulointerstitial fibrosis and poorer renal outcomes, though its prognostic independence remains uncertain (16).

Given these findings, this study aimed to evaluate the reflective and predictive value of urinary podocin, nephrin, and their ratio (PNR) in patients with DKD. By evaluating their temporal association with renal outcomes, this study aims to clarify whether these biomarkers may help identify DKD patients at higher risk of progression. We hypothesized that elevated baseline levels of these biomarkers would correlate with current disease severity (reflected by eGFR and albuminuria) and predict further decline in renal function and worsening albuminuria over a 12-month follow-up.

## 2 Methods

### 2.1 Study design and participants

We conducted a retrospective cohort study with a one-year follow-up involving adult patients with type 2 diabetes and clinically diagnosed DKD. Participants were recruited from nephrology and diabetes outpatient clinics at a tertiary referral hospital between 2023 and 2024. DKD was defined as diabetes with albuminuria and/or reduced eGFR in the absence of other primary kidney diseases, and patients with non-DKD kidney disorders were excluded.

Eligible participants were adults aged 18–70 years with type 2 diabetes, HbA1c <8%, an eGFR >45 mL/min/1.73 m², and albuminuria with a urine albumin-creatinine ratio (uACR) ≥30 mg/g. Additional eligibility required prior participation in a related biomarker study and available baseline urinary podocin, nephrin, and PNR data. Patients were excluded if they had uncontrolled hypertension despite routine ACE inhibitor or angiotensin receptor blocker therapy; acute diabetic complications; recent coronary or cerebrovascular events (within six months); chronic liver disease, malignancy, HIV, systemic infection, malabsorption, or alcohol misuse; a history of smoking; pregnancy or breastfeeding; or participation in other clinical research. Patients with conditions known to cause secondary proteinuria, including urinary tract infection, urolithiasis, or renal tuberculosis, were also excluded. Subjects without retrievable uACR or eGFR data during the follow-up period (9–15 months), or those who discontinued routine renoprotective therapies (ACEi/ARB, SGLT2 inhibitor, GLP-1 receptor agonist, finerenone, or statins), were excluded from analysis. All participants had provided written informed consent permitting the use of their clinical records and biobanked samples. The study protocol was approved by the institutional ethics review board.

At baseline (T0), demographic and clinical characteristics were collected, including age, sex, duration of diabetes, HbA1c, blood pressure, and use of medications known to affect DKD progression (e.g., angiotensin-converting enzyme inhibitors [ACE-I] or angiotensin receptor blockers [ARBs], sodium-glucose cotransporter 2 [SGLT2] inhibitors, statins, and vitamin D analogues). Kidney function was assessed via eGFR, calculated using the CKD-EPI equation, and proteinuria was evaluated using the uACR from a spot urine sample. These served as baseline indicators of renal function and albuminuria.

### 2.2 Urine biomarker measurement

First-morning urine samples were collected at baseline to assess podocyte-specific biomarkers. Urinary podocin levels were determined using the Human Podocin/PDCN (NPHS2) ELISA kit (Abexxa Ltd., Milton, Cambridge, UK), which offers a detection limit of 0.19 ng/mL and a quantifiable range of 0.312–20 ng/mL. Nephrin concentrations were measured using a validated ELISA assay from Exocell (Exocell Inc., Philadelphia, PA, USA), and IL-6 and KIM-1 were assessed using Quantikine^®^ ELISA kits (R&D Systems, Minneapolis, MN, USA). All assays were analytically validated, with documented sensitivity, specificity, linearity, recovery, and intra- and inter-assay precision according to manufacturer specifications.

Samples were processed uniformly—centrifuged to remove cellular debris, aliquoted, and stored at −80 °C until analysis. All ELISA measurements were performed in duplicate, and mean concentrations were used for statistical analysis. The urinary PNR was calculated by dividing podocin concentration by nephrin concentration (both reported in ng/mL), serving as a composite indicator of podocyte structural integrity and injury as described previously (16).

To minimize diurnal variation and fluid intake effects, samples were collected at a consistent early morning time. Creatinine-normalized values were explored but not included in the final analysis as they did not materially alter the results.

### 2.3 Follow-up and outcome definitions

Participants were followed for approximately 12 months. The latest available eGFR and uACR values within this window were defined as endpoint values. Patients without available follow-up laboratory data (either urine or blood tests) within the defined window were excluded from analysis to maintain dataset integrity. Missing values for secondary variables (e.g., metabolic parameters) were not imputed and were analyzed on a complete-case basis. Data completeness was verified prior to statistical analysis to ensure consistency across outcome comparisons.

DKD progression was evaluated using two primary outcomes: decline in kidney function, defined as a decrease in eGFR by ≥5 mL/min/1.73 m² from baseline; or worsening albuminuria, defined as an increase in uACR by ≥30% from baseline. These thresholds were selected based on their use in short-term DKD biomarker and observational studies, where conventional KDIGO criteria requiring ≥40% decline or ESKD events are unlikely to occur within a 12-month interval.

Patients meeting either criterion were categorized as progressors, and those with stable eGFR and uACR were classified as non-progressors. Subgroup analyses were conducted based on age (≥60 *vs*. <60 years), diabetes duration (≥ 10 years *vs*. < 10 years), baseline macroalbuminuria (uACR ≥ 300 mg/g vs. < 300 mg/g) and glycemic control (HbA1c ≥7% vs. <7%) to evaluate whether biomarker performance varied by these risk factors.

### 2.4 Statistical analysis

All statistical analyses were performed using SPSS version 25.0 (IBM Corp., Armonk, NY). Continuous variables were expressed as mean ± standard deviation or median and interquartile range (IQR) depending on distribution, while categorical variables were reported as frequencies and percentages. Group comparisons between progressors and non-progressors used the independent t-test or Mann–Whitney U test for continuous variables and the chi-square test for categorical variables.

To assess the reflective value of urinary podocin, nephrin, and PNR, we examined graphical trends of these biomarkers across different baseline strata of eGFR and albuminuria severity. To analyze biomarker and renal function changes over time, we used the Friedman test to evaluate overall changes in serial measurements (T0, T3, T6, and T12). Where significant, *post-hoc* pairwise comparisons were conducted using the Wilcoxon signed-rank test (e.g., T0 vs. T3, T3 vs. T6).

For graphical representation of longitudinal trends, individual and group-level trajectories of podocin, nephrin, and PNR, as well as eGFR and uACR, were visualized using line plots with error bars (median with IQR). These plots helped illustrate potential temporal patterns and align with both reflective and predictive biomarker interpretation.

To assess predictive value, ROC (Receiver Operating Characteristic) curve analyses were conducted for each biomarker with respect to DKD progression outcomes (eGFR decline and/or uACR increase). The area under the curve (AUC) was calculated to determine predictive performance, with sensitivity and specificity values reported at the optimal cut-off based on Youden’s Index.

All tests were two-tailed with statistical significance set at p < 0.05. A *post-hoc* power analysis was also performed to evaluate whether the sample size was adequate to detect meaningful AUC values.

## 3 Results

### 3.1 Baseline characteristics

A total of 119 patients with DKD were included in the study (**Table 1**). The median age was 59 years (IQR: 52–63), with a slight female predominance (53.78%). Median diabetes duration was 7 years (IQR: 3–13), and 41.53% had diabetes for ≥10 years. The mean HbA1c was 6.94% (SD: 0.69), with 55.46% of participants having suboptimal glycemic control (HbA1c ≥7%).

**Table 1:** Baseline characteristics of study subjects.

Subject characteristics	Total (n = 119)
Age (years), median (IQR)	59 (52–63)
Sex, n (%)	
Male	55 (46.22)
Female	64 (53.78)
Duration of diabetes (years), median (IQR), n = 118	7 (3–13)
Duration of diabetes, n (%), n = 118	
≥ 10 years	49 (41.53)
< 10 years	69 (58.47)
HbA1c, mean (SD)	6.94 (0.69)
HbA1c category, n (%)	
≥ 7%	66 (55.46)
< 7%	53 (44.54)
HDL (mg/dL), median (IQR), n = 57	44 (38–52)
HDL category, n (%), n = 57	
>40 mg/dL in men and >50 mg/dL in women	27 (47.37)
<40 mg/dL in men and <50 mg/dL in women	30 (52.63)
Triglycerides (mg/dL), median (IQR)	147 (108–206)
Triglyceride category, n (%)	
≥ 150 mg/dL	57 (47.90)
< 150 mg/dL	62 (52.10)
ACE inhibitor/ARB therapy, n (%)	
Yes	73 (61.34)
No	46 (38.66)
SGLT2 inhibitor therapy, n (%)	
Yes	5 (4.20)
No	114 (95.80)
Statin therapy, n (%)	
Yes	52 (43.70)
No	67 (56.30)
Calcitriol therapy, n (%)	
Yes	60 (50.42)
No	59 (49.58)
Urine albumin-creatinine ratio (mg/g), median (IQR)	112.0 (40.0–398.17)
Estimated glomerular filtration rate (eGFR) (mL/min/1.73 m²), median (IQR)	68.1 (56–86)
Urinary podocin (ng/mL), median (IQR)	0.51 (0.45–0.62)
Urinary nephrin (ng/mL), median (IQR)	42 (17–250)
Urinary podocin–nephrin ratio, median (IQR)	0.014 (0.002–0.029)

Lipid profile data showed that among the 57 patients with available HDL cholesterol measurements, the median was 44 mg/dL (IQR: 38–52), and 52.63% had levels below sex-specific thresholds. Median triglycerides were 147 mg/dL (IQR: 108–206), with 47.90% exceeding 150 mg/dL.

Regarding pharmacologic treatment, 61.34% were on ACE inhibitors or ARBs, while only 4.20% were receiving SGLT2 inhibitors. Statins were used by 43.70%, and 50.42% were on calcitriol.

At baseline, the median uACR was 112.0 mg/g (IQR: 40.0–398.17). A detailed classification showed that 73.9% of subjects had moderately increased albuminuria (microalbuminuria), while 26.1% had severely increased albuminuria (macroalbuminuria). The median eGFR was 68.1 mL/min/1.73 m² (IQR: 56–86). Overall, 66.4% of subjects had eGFR >60 mL/min/1.73 m², and 33.6% fell within the 45–60 mL/min/1.73 m² range, corresponding to CKD stages 2 and 3a, respectively.

Podocyte-specific biomarkers showed a median urinary podocin level of 0.51 ng/mL (IQR: 0.45–0.62), nephrin of 42 ng/mL (IQR: 17–250), and a PNR of 0.014 (IQR: 0.002–0.029).

### 3.2 Changes in clinical and biomarker parameters over 12 months

**Figure 1:**
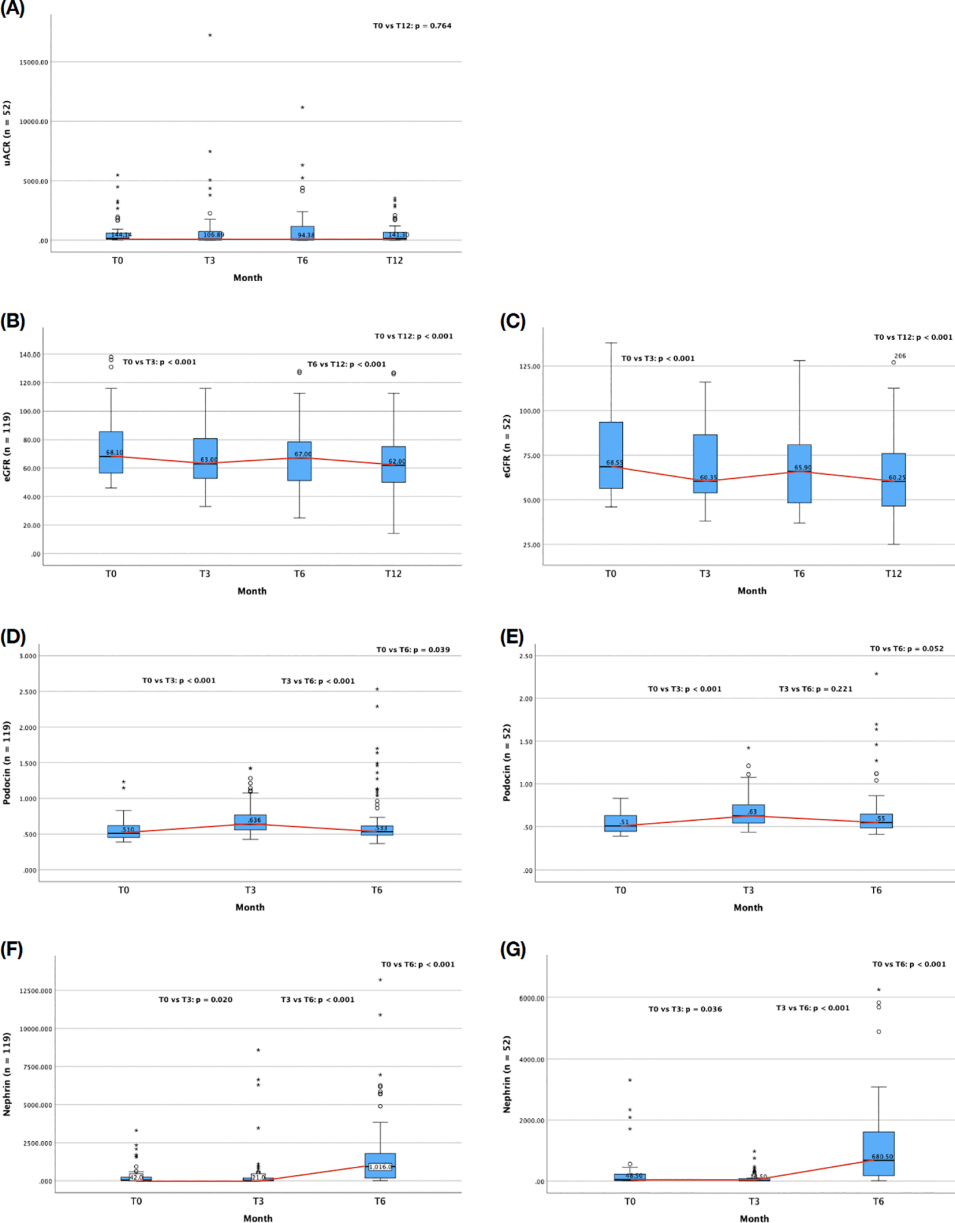
Trends in urinary biomarkers and renal function over 12 months of follow-up. Median values with interquartile ranges are shown at baseline (T0), 3 months (T3), 6 months (T6), and 12 months (T12). **(A)** Trend of median uACR changes over one year of follow-up (n = 52) **(B)** Trend of median eGFR changes over one year of follow-up (n = 119) **(C)** Trend of median eGFR changes over one year of follow-up (n = 52) **(D)** Trend of median urinary podocin levels over one year of follow-up (n = 119) **(E)** Trend of median urinary podocin levels over one year of follow-up (n = 52) **(F)** Trend of median urinary nephrin levels over one year of follow-up (n = 119) **(G)** Trend of median urinary nephrin levels over one year of follow-up (n = 52). Overall longitudinal changes were assessed using the Friedman test. Pairwise comparisons between time points were performed using the Wilcoxon signed-rank test. Significant pairwise differences are indicated in the figure. Non-significant comparisons are not shown.

At the end of the 12-month follow-up, uACR data were available for 52 patients. Among patients with complete follow-up data, uACR showed a fluctuating pattern. Median uACR decreased slightly from 106.89 mg/g at 3 months (T3) to 94.38 mg/g at 6 months (T6), but increased again to 141.3 mg/g by 12 months (T12). These variations were not statistically significant across any time points, including from baseline to T12 (Z = –0.301; p = 0.764), indicating high inter-individual variability and possible episodic glomerular injury. This pattern is visually represented in **Figure 1A**, which illustrates the temporal variability of uACR.

In contrast, eGFR showed a clearer trend of progressive decline. In the overall cohort (n = 119), median eGFR decreased from 68.1 to 62.0 mL/min/1.73 m² over 12 months. This decline was statistically significant from T0 to T3 (Z = –4.373; p < 0.001) and from T6 to T12 (Z = –4.014; p < 0.001), but not from T3 to T6 (p = 0.835), suggesting a potential period of transient stabilization. Similar patterns were observed in the subgroup with complete uACR data (n = 52), with a significant drop from baseline to 12 months (Z = –4.267; p < 0.001). These trends are illustrated in **Figures 1B, C**, showing the trajectory of eGFR decline over time.

Urinary podocin levels also changed significantly over time (**Figures 1D, E**). Median podocin increased from 0.51 ng/mL at baseline to 0.636 ng/mL at T3 (Z = –**7**.565; p < 0.001), followed by a decline to 0.533 ng/mL at T6 (Z = –3.392; p < 0.001), suggesting initial podocyte stress with subsequent possible detachment or loss.

Nephrin levels displayed a more volatile trajectory (**Figures 1F, G**). Median urinary nephrin dropped from 42 ng/mL to 21 ng/mL at T3 (Z = –2.322; p = 0.020), followed by a sharp rise to 1016 ng/mL at T6 (Z = –9.184; p < 0.001), indicating a surge in podocyte injury. These trends were also significant in the subgroup with full follow-up data.

### 3.3 Progression outcomes and predictive value of biomarkers

All patients were followed for a median duration of 12 months (range: 9–15 months). However, final uACR data were available for only 52 subjects at the end of follow-up. During this period, renal outcomes varied: some patients maintained stable kidney function or showed slight improvement (likely due to optimized management), while others experienced progression. Based on predefined criteria, approximately one-third of the cohort demonstrated DKD progression. Specifically, 19 patients (36.5%) exhibited a decline in eGFR ≥5 mL/min/1.73 m², and 17 patients (32.7%) showed an increase in uACR ≥30%. Ten patients (19%) met both criteria, indicating concordant worsening of renal function and albuminuria. Importantly, this overlap was reported descriptively only, and no combined (composite) endpoint was used for statistical analysis. No patient progressed to end-stage kidney disease or required dialysis during the study period.

To assess the predictive value of podocyte biomarkers, baseline levels of podocin, nephrin, and the PNR were compared between progressors and non-progressors. Median urinary nephrin was higher among progressors (~60 ng/mL) compared to non-progressors (~45 ng/mL); podocin was slightly higher as well (0.53 vs. 0.50 ng/mL). PNR also trended higher in the progression group (0.013 vs. 0.010). However, none of these differences reached statistical significance (all p > 0.3, Mann–Whitney U test), and there was substantial overlap between groups, suggesting limited discriminatory value of baseline biomarker levels for short-term progression.

**Figure 2:**
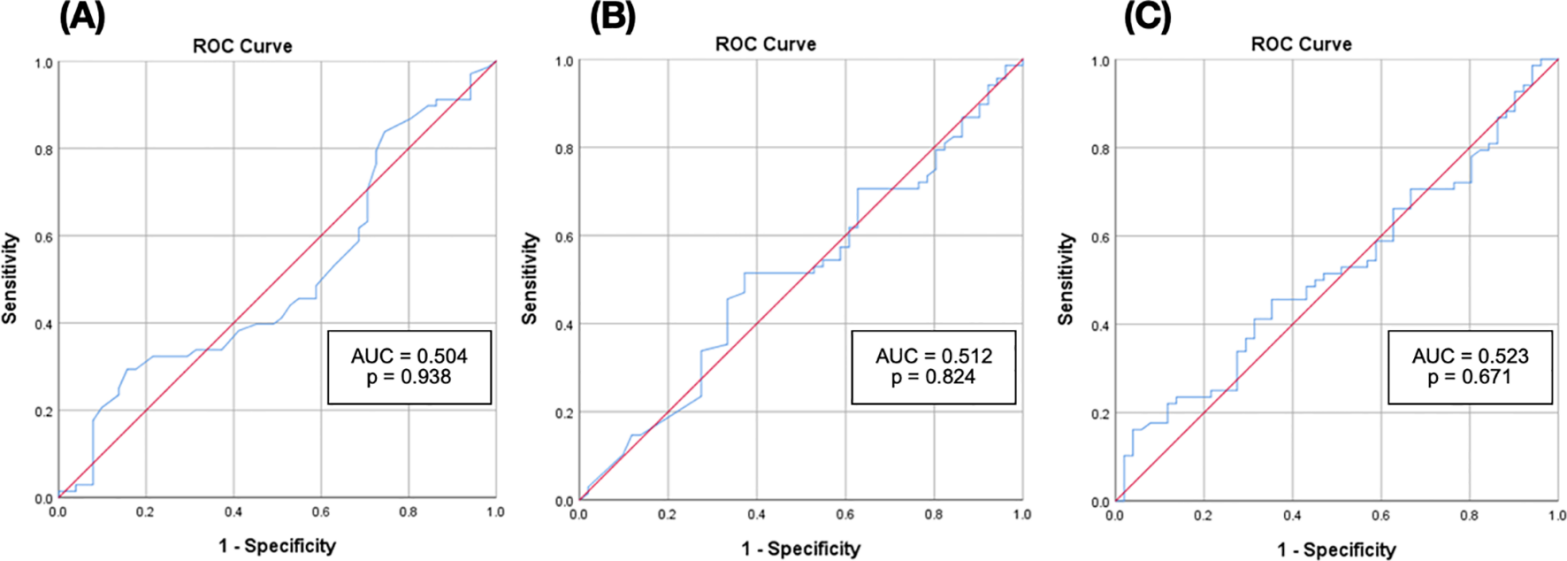
Receiver operating characteristic (ROC) curves of urinary biomarkers predicting decline in eGFR. **(A)** ROC curve of podocin for predicting a decline in eGFR ≥ 5 mL/min; n = 119 (p = 0.938) **(B)** ROC curve of nephrin for predicting a decline in eGFR ≥ 5 mL/min; n = 119 (p = 0.824) **(C)** ROC curve of the PNR for predicting a decline in eGFR ≥ 5 mL/min; n = 119 (p = 0.671).

ROC analysis further evaluated the biomarkers’ predictive performance. For eGFR decline, the AUCs were: podocin 0.504 (95% CI: 0.398–0.610, p = 0.94), nephrin 0.512 (CI: 0.407–0.617, p = 0.82), and PNR 0.523 (CI: 0.418–0.627, p = 0.67) (**Figures 2A–C**). These values approximated 0.5, indicating no predictive capacity beyond chance.

**Figure 3:**
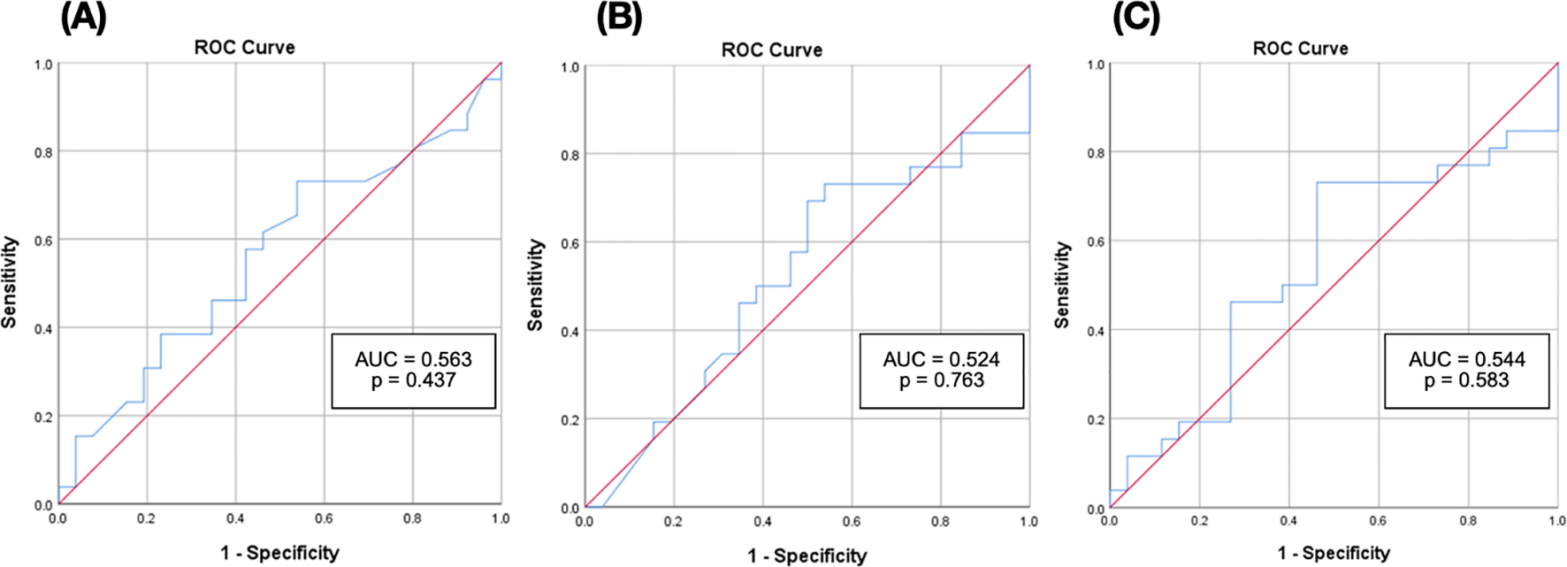
Receiver operating characteristic (ROC) curves of urinary biomarkers predicting increase in uACR. **(A)** ROC curve of podocin for predicting an increase in uACR ≥ 30%; n = 52 (p = 0.437) **(B)** ROC curve of nephrin for predicting an increase in uACR ≥ 30%; n = 52 (p = 0.763) **(C)** ROC curve of the PNR for predicting an increase in uACR ≥ 30%; n = 52 (p = 0.583).

For predicting albuminuria progression, AUC values were slightly higher but still non-significant: podocin 0.563 (CI: 0.405–0.721, p = 0.44), nephrin 0.524 (CI: 0.364–0.685, p = 0.76), and PNR 0.544 (CI: 0.383–0.706, p = 0.58) (**Figures 3A–C**). While PNR showed the highest AUC among the three, the difference was minimal and not statistically meaningful.

A logistic regression model combining podocin and nephrin also failed to improve discrimination, yielding an AUC of 0.55—comparable to PNR alone. This suggests that combining the two markers did not provide added predictive value in this cohort.

Subgroup analyses were conducted based on HbA1c ≥ 7%, diabetes duration ≥ 10 years, age ≥ 60 years, and baseline macroalbuminuria (uACR ≥ 300 mg/g). Across all subgroups, urinary podocin, nephrin, and the PNR showed limited predictive value for both eGFR decline and uACR increase over 12 months. None of the ROC analyses reached statistical significance (all p > 0.05), and AUC values generally ranged from poor to fair discrimination. Detailed AUCs with 95% confidence intervals and p-values, including are presented in **Supplementary Table 1** and **Supplementary Figures 1**-**8**).

A *post-hoc* power analysis was performed for the ROC/AUC analyses using the Hanley & McNeil approximation (two-sided α = 0.05). Given the observed AUCs and event counts (eGFR decline: 19 events/119; uACR increase: 17 events/52), the statistical power to detect the observed AUCs as different from 0.5 was low (range 0.05–0.16 for eGFR analyses and 0.06–0.14 for uACR analyses).

## 4 Discussion

This retrospective cohort study assessed the prognostic value of urinary podocin, nephrin, and PNR in predicting short-term DKD progression over one year. Our results indicate that none of these biomarkers, either individually or in combination, demonstrated adequate predictive performance for significant decline in eGFR or worsening albuminuria. ROC analysis revealed modest AUC values, all below 0.60, and none reached statistical significance, suggesting that a single baseline measurement of urinary podocin, nephrin, or their ratio lacks sufficient discriminatory power for short-term risk stratification in DKD.

These findings contrast with previous studies reporting strong associations between podocyte-specific biomarkers and DKD severity. Rather than confirming earlier optimism from cross-sectional studies, our results suggest a more conservative interpretation of their clinical role. Prior work has shown that urinary podocin and nephrin correlate with albuminuria severity and may distinguish individuals with DKD from those without disease; however, most of those studies were diagnostic rather than prognostic in design and compared groups with large biological contrasts. For example, ElShaarawy et al. (18) reported an AUC of 0.977 for urinary podocin in differentiating early DKD, while studies by Kostovska et al. (4) and Kondapi et al. (10) demonstrated high diagnostic accuracy for urinary nephrin across albuminuria stages. However, these studies predominantly utilized cross-sectional or case-control designs comparing individuals with and without DKD, which may have exaggerated diagnostic performance due to greater intergroup contrasts. Our longitudinal design, in contrast, focused on prognostic assessment within a more clinically homogeneous DKD population, offering a more stringent test of biomarker utility.

By focusing on a relatively homogeneous cohort already diagnosed with DKD and following them longitudinally, this investigation provides a more rigorous test of biomarker utility. The weak discriminatory performance observed here suggests that podocyte-derived proteins may function better as reflective indicators of ongoing injury rather than forward-looking predictors of functional decline—at least when assessed as a single time-point measurement.

Despite the lack of significant predictive value, our findings reaffirm previous observations that urinary podocin and nephrin are detectable in patients with DKD and are numerically elevated in those with longer diabetes duration or poorer glycemic control. These trends, although not statistically significant in our cohort, align with prior reports that associate elevated podocyte-specific proteins with glomerular stress and structural injury. Jim et al. (7) and Fukuda et al. (11), for instance, found elevated urinary nephrin and podocin levels in patients with overt DKD, while Kondapi et al. (10) noted podocinuria even in normoalbuminuric diabetics with metabolic risk factors. Similarly, PNR demonstrated numerically better—but still insufficient—performance for detecting albuminuria progression, aligning with mechanistic hypotheses suggesting that disproportionate podocin elevation may indicate more advanced structural injury. These trends reinforce the biological relevance of podocyte injury pathways in DKD, even though the markers did not translate into actionable prognostic tools in this timeframe.

Importantly, our results align with those of Zeng et al., who investigated the urinary PNR mRNA ratio in biopsy-proven DKD and found only modest prognostic value for short-term renal outcomes. Although a higher PNR was associated with histologic fibrosis, it did not retain independent prognostic significance after multivariate adjustment (16). In our study, the protein-level PNR showed slightly better performance than individual markers for predicting albuminuria progression but remained below the threshold for clinical utility. This suggests that while PNR may biologically reflect greater podocyte injury, it is not yet a robust standalone predictor of disease progression over a 12-month timeframe.

In contrast, earlier studies reported much higher discriminatory accuracy—though mostly in diagnostic rather than prognostic contexts. For instance, ElShaarawy et al. (2019) (18) reported an AUC of 0.977 for urinary podocin in distinguishing DKD from non-DKD diabetics, while Veluri et al. (2022) (3) found urinary nephrin to have 100% sensitivity and 88% specificity for detecting overt DKD. Kostovska et al. similarly reported a 96% predicted probability of nephrinuria for identifying nephropathy (4). However, these studies aimed to differentiate established disease from healthy or at-risk states, not to predict disease progression within already affected individuals. The narrower dynamic range and subtler within-group differences in our cohort may explain the weaker prognostic performance.

Some authors have proposed that PNR, whether at the mRNA or protein level, may offer a more integrated signal of podocyte damage than individual markers. Fukuda et al. (17) and Zeng et al. (16) found that a higher PNR may reflect more advanced structural injury—given that nephrin loss may occur early through slit diaphragm disruption, whereas podocin loss could indicate deeper cytoskeletal damage or podocyte detachment. This biological rationale supports the hypothesis that a rising PNR may indicate worsening glomerular integrity.

However, the combination of urinary podocin and nephrin also failed to provide additional predictive value in our study. This may be explained by their distinct biological characteristics and temporal expression patterns in relation to podocyte injury. Podocin, a podocyte membrane protein, when detected in urine, may reflect podocyte detachment or shedding (19). An elevated urinary podocin level (podocinuria) suggests greater podocyte loss and has been proposed as a surrogate for faster GFR decline, as supported by the “podocyte depletion hypothesis” in DKD (11). This hypothesis posits that ongoing podocyte loss is a central mechanism driving DKD progression, which may be captured by rising podocin excretion.

In contrast, urinary nephrin levels tend to rise in the early phases of DKD—even before microalbuminuria—indicating initial podocyte stress or foot process effacement. However, nephrinuria may plateau or decline in advanced disease stages. Once overt proteinuria occurs, albuminuria itself may serve as an equally strong indicator of progression risk compared to nephrin (11). Furthermore, nephrin (a large extracellular protein) may undergo urinary degradation or be subject to tubular reabsorption, which could affect its stability and signal consistency. These factors may explain why urinary nephrin mRNA was not found to be independently predictive of short-term outcomes in the study by Zeng et al., and why nephrin-based biomarkers often fail to significantly enhance predictive models beyond albuminuria (16).

Moreover, the podocytopathy in DKD evolves over time. Initially, podocyte stress leads to increased nephrin excretion. As damage progresses, nephrin expression wanes, and podocin, while elevated at first, may also eventually decline. These dynamic changes suggest that neither podocin nor nephrin levels remain consistently elevated across disease stages. Consequently, while both biomarkers may serve as useful indicators of early glomerular injury or active damage, they appear less suitable for long-term prognostication of renal function decline. This aligns with our findings, where neither individual nor combined biomarker measurements significantly predicted eGFR changes or albuminuria progression after one year.

The rationale for exploring the PNR was to assess qualitative shifts in podocyte phenotype. A disproportionately higher podocin relative to nephrin may indicate podocyte hypertrophy or structural reorganization under stress (20). Indeed, previous studies have shown that a higher PNR correlates with greater histologic damage (glomerulosclerosis, interstitial fibrosis) in DKD patients (11, 17, 20). This suggests that an imbalance in slit diaphragm protein expression reflects more advanced glomerular injury. However, the prognostic utility of this ratio remains limited.

In our study, although PNR was higher in progressors and had the best AUC among the three markers for predicting worsening albuminuria, the improvement was minimal and non-significant. It is plausible that a longer follow-up duration might allow PNR differences to widen as structural damage becomes clinically apparent. As such, while our data support the potential biological plausibility of PNR as a risk marker, they also underscore its limited short-term prognostic utility in clinical practice.

Taken together, these results contribute novel evidence that urinary podocin, nephrin, and PNR—despite strong mechanistic justification—are not yet suitable standalone predictors for short-term DKD progression when measured once at baseline. The study shifts the current understanding from biomarker enthusiasm toward a more cautious position and highlights the need for future research focusing on repeated measurements, longer follow-up, multimarker models, or integration with clinical risk scores or omics-based signatures. Such approaches may determine whether the prognostic value of podocyte biomarkers lies not in absolute levels, but in longitudinal behavior, thresholds of change, or interaction with other pathways.

## 5 Limitations and strengths

This study should be interpreted considering certain limitations. The sample size, while modest, may have reduced the statistical power to detect subtle associations between urinary biomarkers and clinical outcomes. *Post-hoc* power analysis demonstrated that the statistical power of the ROC analyses was low (range 0.05–0.16), largely due to the limited number of progression events. Detecting an AUC in the range of 0.8—as reported in previous studies—would require approximately 80 participants, with even larger cohorts necessary to detect the more modest AUC values observed here (~0.6). The resulting wide confidence intervals in our ROC analyses suggest that smaller, yet potentially meaningful, effects could not be definitively confirmed in this cohort. Additionally, the one-year follow-up duration, although practical for clinical monitoring, may have limited our ability to capture more pronounced progression events, especially among individuals with preserved eGFR or only microalbuminuria at baseline.

Another important limitation is the significant loss of follow-up for uACR measurements, with only 52 participants completing the 12-month evaluation. This degree of attrition introduces a risk of selection and information bias. Participants who did not complete follow-up may have had different clinical characteristics, disease trajectories, or treatment responses compared to those retained in the analysis. As such, the observed associations between urinary biomarkers and renal outcomes may underestimate or overestimate true effects. Future studies with more robust strategies for handling missing data—such as multiple imputation or inverse probability weighting—are warranted to reduce bias and strengthen validity.

Despite these constraints, this study offers several strengths. It is among the first to assess the podocin-nephrin ratio (PNR) at the protein level in a clinical cohort of patients with diabetic kidney disease, using a retrospective design and first-morning urine samples to reduce biological and analytical variability. The cohort was well-characterized, allowing contextual interpretation of biomarker values relative to baseline renal function and albuminuria severity. Furthermore, focusing on a homogenous DKD population—rather than mixed CKD etiologies—enhances clinical relevance and translational applicability. Importantly, the negative and borderline findings reported here contribute meaningfully to the literature by preventing publication bias and providing realistic expectations regarding early biomarker performance. Transparent reporting of these results underscores the need for cautious interpretation of early-phase biomarker research and supports ongoing efforts toward standardized, longitudinal, and evidence-based biomarker validation in DKD.

## 6 Future directions

Larger, multicenter studies with longer follow-up are needed to determine whether serial measurements or temporal shifts in podocin, nephrin, or their ratio can predict long-term DKD progression and treatment response. Further mechanistic research should clarify how podocyte injury—characterized by cytoskeletal disruption, slit diaphragm destabilization, and cellular detachment—leads to urinary podocyte protein release. Understanding these pathways may help identify therapeutic targets and support the use of podocyte-derived biomarkers as pharmacodynamic indicators in evaluating emerging podocyte-protective therapies and biomarker-guided drug discovery efforts.

## 7 Conclusion

In this 12-month retrospective cohort study, urinary podocin, nephrin, and PNR did not demonstrate adequate predictive value for short-term DKD progression, as measured by decline in eGFR or worsening albuminuria. Although elevated levels of these biomarkers may reflect underlying podocyte injury and glomerular stress, their ability to independently predict renal outcomes was limited. These findings highlight the need for further longitudinal research with larger cohorts and serial biomarker assessments to better define the prognostic utility of podocyte-derived proteins in DKD.

## Data Availability

The raw data supporting the conclusions of this article will be made available by the authors, without undue reservation.
