# Supplementary material for: Reflective versus predictive value of urinary podocin, nephrin, and their ratio in diabetic kidney disease: a 12-month retrospective cohort study
Source: Front Nephrol. 2026 Jan 21;5:1681679. doi: 10.3389/fneph.2025.1681679 (PMC12867881; doi:10.3389/fneph.2025.1681679)
Supplement: Supplementary file 1 [file DataSheet1.docx]

Supplementary Material

# Supplementary Figures and Tables

## Supplementary Tables

**Supplementary Table 1:** AUCs of Urinary Podocin, Nephrin, and Podocin-Nephrin Ratio (PNR) for Predicting eGFR Decline and uACR Increase Across Subgroups

| **Subgroup** | **Outcome** | **Biomarker** | **AUC (95% CI)** | **p-value** |
| --- | --- | --- | --- | --- |
| **HbA1c ≥ 7% (n = 66 for eGFR, n = 27 for uACR)** | eGFR decline | Podocin | 0.520 (0.373–0.668) | 0.785 |
|  |  | Nephrin | 0.504 (0.357–0.651) | 0.957 |
|  |  | PNR | 0.529 (0.385–0.672) | 0.699 |
|  | uACR increase | Podocin | 0.542 (0.312–0.772) | 0.714 |
|  |  | Nephrin | 0.453 (0.228–0.678) | 0.678 |
|  |  | PNR | 0.589 (0.361–0.817) | 0.435 |
| **Diabetes duration ≥ 10 years (n = 49 for eGFR, n = 27 for uACR)** | eGFR decline | Podocin | 0.454 (0.287–0.621) | 0.600 |
|  |  | Nephrin | 0.545 (0.374–0.716) | 0.607 |
|  |  | PNR | 0.450 (0.279–0.622) | 0.571 |
|  | uACR increase | Podocin | 0.484 (0.249–0.719) | 0.892 |
|  |  | Nephrin | 0.490 (0.254–0.727) | 0.935 |
|  |  | PNR | 0.513 (0.276–0.750) | 0.913 |
| **Age ≥ 60 years (n = 54 for eGFR, n = 21 for uACR)** | eGFR decline | Podocin | 0.478 (0.319–0.637) | 0.781 |
|  |  | Nephrin | 0.483 (0.327–0.640) | 0.835 |
|  |  | PNR | 0.517 (0.361–0.673) | 0.828 |
|  | uACR increase | Podocin | 0.491 (0.235–0.746) | 0.944 |
|  |  | Nephrin | 0.441 (0.181–0.701) | 0.647 |
|  |  | PNR | 0.582 (0.322–0.842) | 0.526 |
| **Baseline uACR ≥ 300 mg/g (n = 32 for eGFR, n = 20 for uACR)** | eGFR decline | Podocin | 0.477 (0.254–0.700) | 0.839 |
|  |  | Nephrin | 0.695 (0.503–0.888) | 0.080 |
|  |  | PNR | 0.309 (0.121–0.497) | 0.088 |
|  | uACR increase | Podocin | 0.643 (0.386–0.900) | 0.303 |
|  |  | Nephrin | 0.478 (0.198–0.758) | 0.874 |
|  |  | PNR | 0.516 (0.230–0.803) | 0.905 |

## Supplementary Figures


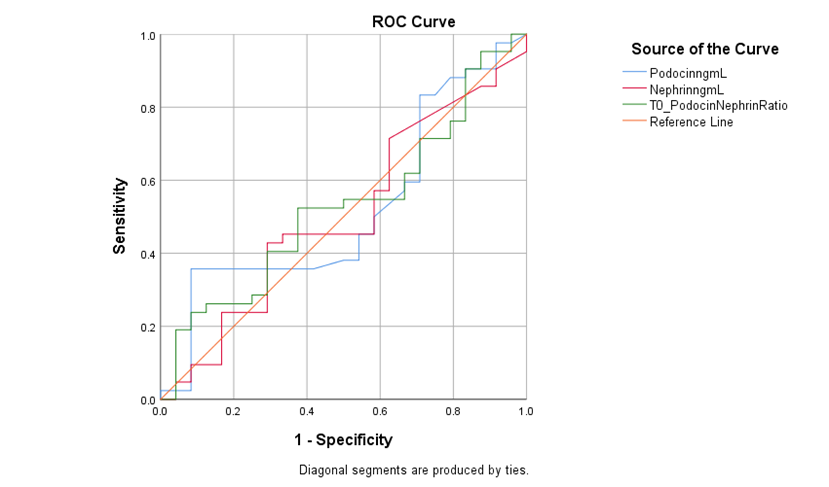


**Supplementary Figure 1.** ROC curves of podocin, nephrin, and the podocin-nephrin ratio for detecting ≥5 mL/min/1.73 m² eGFR decline in subjects with HbA1c ≥ 7%; n = 66 (p = 0.785, 0.957, and 0.699, respectively)

**
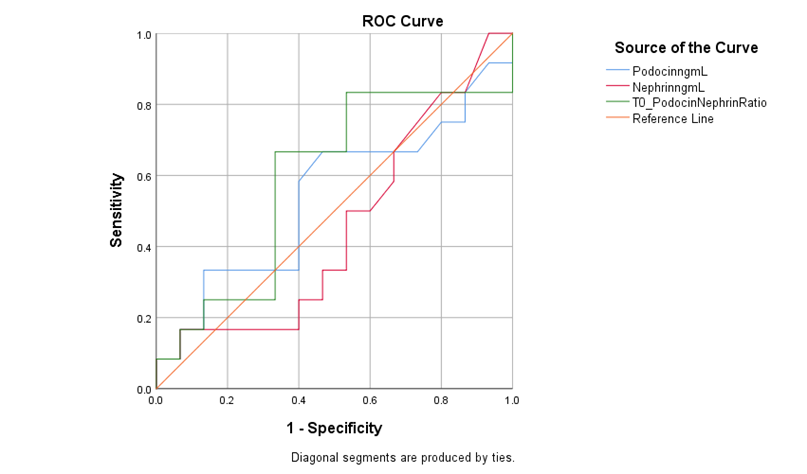
**

**Supplementary Figure 2.** ROC curves of podocin, nephrin, and the podocin-nephrin ratio for detecting ≥30% increase in uACR in subjects with HbA1c ≥ 7%; n = 27 (p = 0.714, 0.678, and 0.435, respectively)


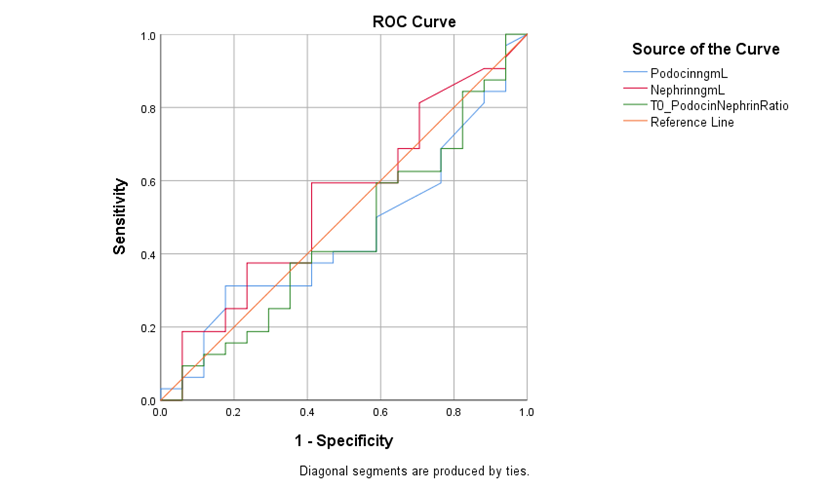


**Supplementary Figure 3.** ROC curves of podocin, nephrin, and the podocin-nephrin ratio for detecting ≥5 mL/min/1.73 m² eGFR decline in subjects with diabetes duration ≥ 10 years; n = 49 (p = 0.600, 0.607, and 0.571, respectively)

**
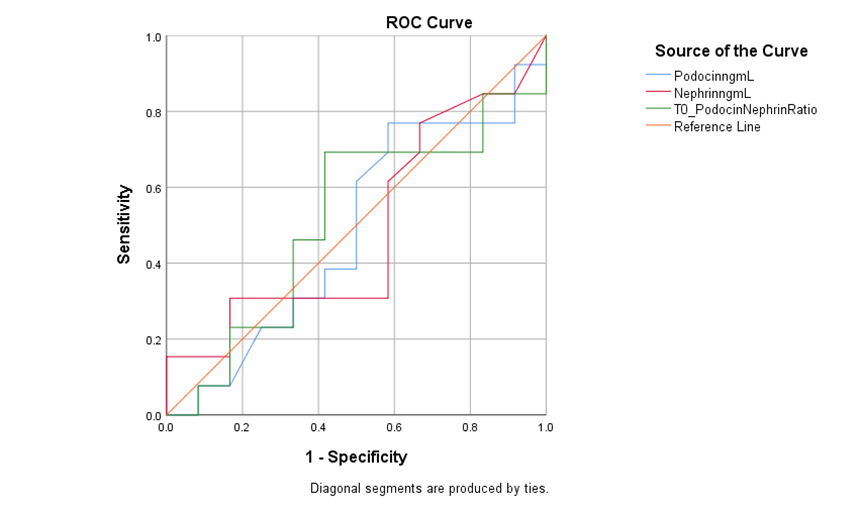
**

**Supplementary Figure 4.** ROC curves of podocin, nephrin, and the podocin-nephrin ratio for detecting ≥30% increase in uACR in subjects with diabetes duration ≥ 10 years; n = 27 (p = 0.892, 0.935, and 0.913, respectively)


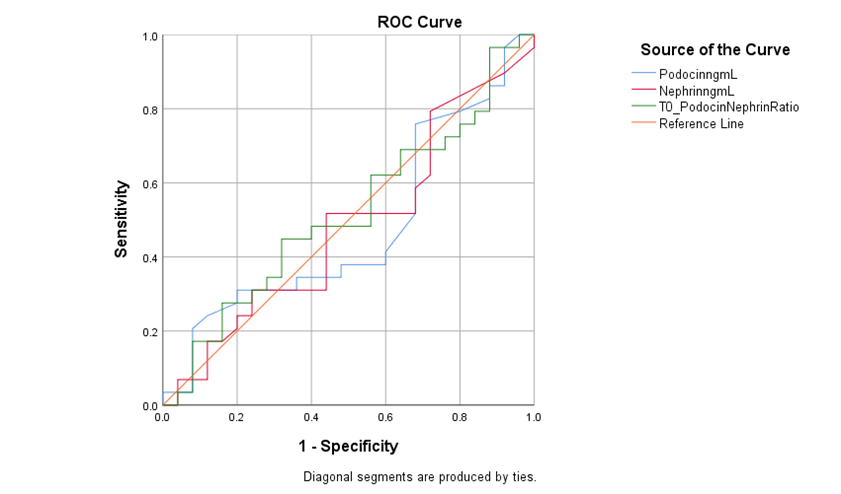


**Supplementary Figure 5.** ROC curves of podocin, nephrin, and the podocin-nephrin ratio for detecting ≥5 mL/min/1.73 m² eGFR decline in subjects aged ≥ 60 years; n = 54 (p = 0.781, 0.835, and 0.828, respectively)

**
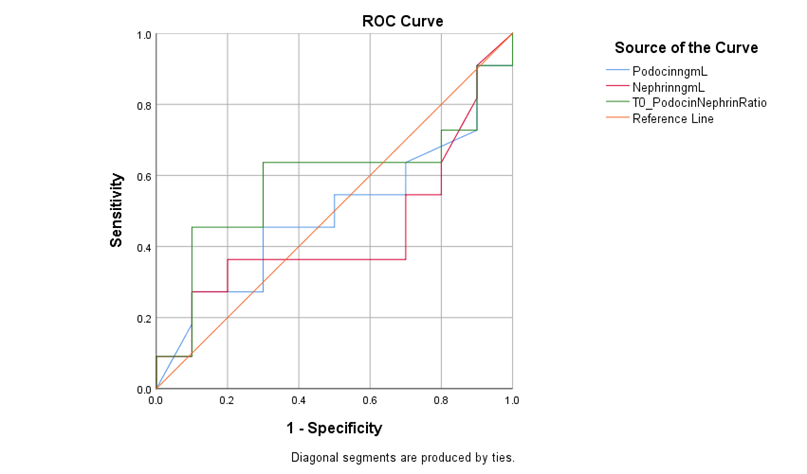
**

**Supplementary Figure 6.** ROC curves of podocin, nephrin, and the podocin-nephrin ratio for detecting ≥30% increase in uACR in subjects aged ≥ 60 years; n = 21 (p = 0.944, 0.647, and 0.526, respectively)


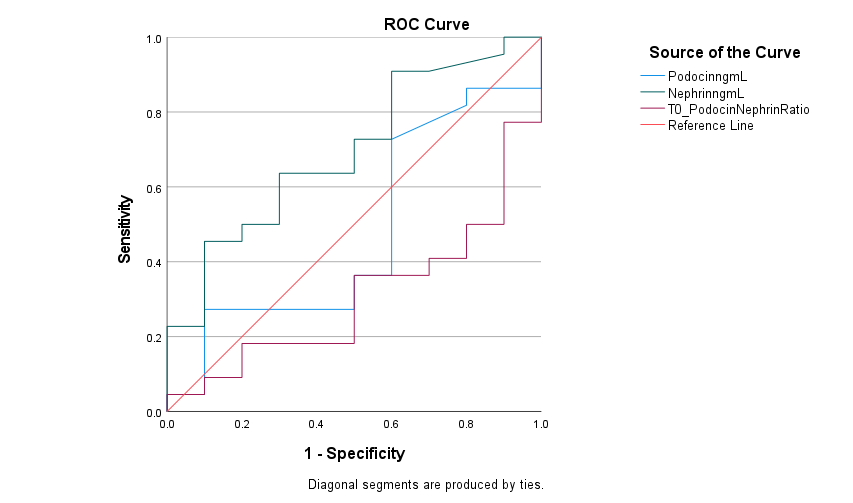


**Supplementary Figure 7.** ROC curves of podocin, nephrin, and the podocin-nephrin ratio for detecting ≥5 mL/min/1.73 m² eGFR decline in subjects with baseline uACR ≥ 300 mg/g; n = 32 (p = 0.839, 0.080, and 0.088, respectively)

**
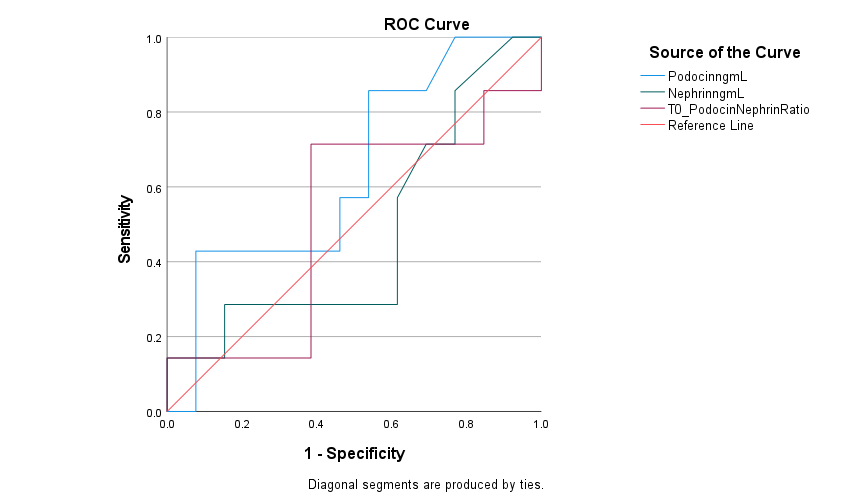
**

**Supplementary Figure 8.** ROC curves of podocin, nephrin, and the podocin-nephrin ratio for detecting ≥30% increase in uACR in subjects with baseline uACR ≥ 300 mg/g; n = 20 (p = 0.303, 0.874, and 0.905, respectively)
